# Estimating Risk of Circulatory Disease: Little et al. Respond

**DOI:** 10.1289/ehp.1206046R

**Published:** 2012-12-03

**Authors:** Mark P. Little, Dimitry Bazyka, Simon D. Bouffler, John D. Harrison, Elisabeth Cardis, Francis A. Cucinotta, Michaela Kreuzer, Olivier Laurent, Soile Tapio, Richard Wakeford, Lydia Zablotska, Steven E. Lipshultz

**Affiliations:** Radiation Epidemiology Branch, National Cancer Institute, Rockville, Maryland, E-mail: mark.little@nih.gov; Research Center for Radiation Medicine, Kyiv, Ukraine; Health Protection Agency, Chilton, United Kingdom; CREAL (Center for Research in Environmental Epidemiology), Spain; Radiation Health Office, NASA Johnson Space Center, Houston, Texas; Department of Radiation Protection and Health, Federal Office for Radiation Protection, Oberschleissheim, Germany; Laboratoire d’Epidémiologie, Institut de Radioprotection et de Sûreté Nucleaire, Fontenay-aux-Roses, France; Helmholtz Zentrum München, Institute of Radiation Biology, Oberschleissheim, Germany; Dalton Nuclear Institute, University of Manchester, Manchester, United Kingdom; Department of Epidemiology and Biostatistics, University of California San Francisco, San Francisco, California; Department of Pediatrics, Leonard M. Miller School of Medicine, University of Miami, Miami, Florida

We welcome Schöllnberger and Kaiser’s comments on our review (Little et al. 2012). The biology of radiation-associated athero-sclerosis has been extensively reviewed (Advisory Group on Ionising Radiation 2010; Little et al. 2010). As we stated in our paper, there are “biological data suggesting that many inflammatory end points potentially relevant to circulatory disease may be differentially regulated below and above about 0.5 Gy,” which is why we studied low-to-moderate exposures (Little et al. 2012). Mitchel et al. (2011) and Rödel et al. (2012) support a possible biphasic dose response, as do many other data (Advisory Group on Ionising Radiation 2010; Little et al. 2010).

Schöllnberger et al. used multi-model inference (Burnham and Anderson 1998) to assess circulatory disease risk in their analysis of the Life Span Study (LSS) cohort of atomic-bomb survivors who were exposed briefly to radiation (Schöllnberger et al. 2012). We doubt that the effect they observed can be simply generalized to studies of other groups, in particular those chronically exposed. More important, most studies do not have information on potential confounders. We judge that the focus should not be to improve statistical modeling techniques, but to critically address the problems of confounding or other bias and to assess low-dose biological mechanisms.

We also question the validity of the threshold models Schöllnberger et al. (2012) used. No data suggest a threshold for biological markers relevant to circulatory disease (Advisory Group on Ionising Radiation 2010; Little et al. 2010).

Schöllnberger et al. (2012) used older LSS data (Preston et al. 2003) limited to deaths in proximal survivors since 1968; we judge these restrictions to be questionable for circulatory disease end points. In our analyses (Little et al. 2012), we used current LSS data (Shimizu et al. 2010) that show substantially more deaths (12,139 vs. 3,954 for stroke; 14,018 vs. 4,477 for heart diseases), which means the analysis by Schöllnberger et al. (2012) has much less statistical power and that some of their inferences are likely inconsistent with the current data.

In summary, Schöllnberger et al. (2012)used biologically questionable models fitted to a single, older (LSS) data set, disregarding evidence from radiation-induced circulatory disease risks in several populations with low-to-moderate exposures (Little et al. 2012). It is important to know whether low doses or dose rates of radiation are associated with increased morbidity and premature mortality and, if so, by what mechanism. The point of our paper was to address this clinical and public health concern.
